# A Pomegranate Polyphenol Extract Suppresses the Microbial Production of Proatherogenic Trimethylamine (TMA) in an In Vitro Human Colon Model

**DOI:** 10.1002/mnfr.70166

**Published:** 2025-06-29

**Authors:** Julia E. Haarhuis, Priscilla Day‐Walsh, Emad Shehata, George M. Savva, Barbora Peck, Mark Philo, Paul A. Kroon

**Affiliations:** ^1^ Quadram Institute Bioscience, Norwich Research Park Norwich UK; ^2^ Department of Obstetrics and Gynaecology University of Cambridge, The Rosie Hospital Robinson Way Cambridge UK; ^3^ Centre for Trophoblast Research (CTR), Department of Physiology, Development and Neuroscience University of Cambridge Cambridge UK; ^4^ Chemistry of Flavour and Aroma Department Food Industry and Nutrition Research Institute, National Research Centre Dokki Cairo Egypt

**Keywords:** anaerobic fermentation, ellagitannins, gut microbiota metabolism, punicalagin, trimethylamine *N*‐oxide

## Abstract

High circulating levels of trimethylamine *N*‐oxide (TMAO) are linked to metabolic diseases, adverse outcomes after heart failure, and atherogenic effects in animal models and in human subjects. l‐Carnitine and choline are major dietary precursors of TMAO. These are first converted to trimethylamine (TMA) by gut microbiota, which is absorbed by the host and converted into TMAO by hepatic flavin‐containing monooxygenases (FMOs). The minimal absorption of pomegranate polyphenols by the host suggests that they may reach the colon for further metabolism by the gut microbiome. This study investigates the ability of a polyphenol‐rich pomegranate extract to inhibit TMA production by human fecal microbiota. Batch fermentations were conducted with 1% human fecal inoculum, l‐carnitine, or choline, and a pomegranate extract (anaerobic, pH 6.6–7.1, 37°C) for 24 or 48 h. Methylamines were quantified using LC‐MS/MS with isotopically labeled internal standards. The pomegranate extract significantly delayed and reduced the rate of TMA production from both choline and l‐carnitine. The effect was dose‐dependent for l‐carnitine, with the highest dose delaying the average midpoint of l‐carnitine metabolism by 16 h (95% CI = 8.4‐24; *p* = 0.001). The pomegranate extract significantly reduced TMA production from choline and l‐carnitine in vitro.

## Graphical abstract

Trimethylamine *N*‐oxide (TMAO) is associated with various cardiometabolic and other diseases and can cause thrombosis and vascular inflammation. Dietary l‐carnitine and choline are metabolized by gut microbiota to trimethylamine (TMA), which is oxidized to TMAO in the liver. This study shows that a pomegranate extract significantly inhibits the conversion of l‐carnitine and choline to TMA by gut microbiota.

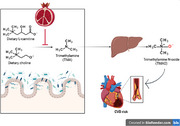

**Abbreviations**
γ‐BB: γ‐butyrobetaine
CFU: colony‐forming unit
DMB: 3,3‐dimethyl‐1‐butanol
FMO: flavin‐containing monooxygenase
IC_50_
: half‐maximal inhibitory concentration
MSC: microbiological safety cabinet
RT: retention time
TMA: trimethylamine
TMAO: Trimethylamine *N*‐oxide

## 1 Introduction

**FIGURE 1:**
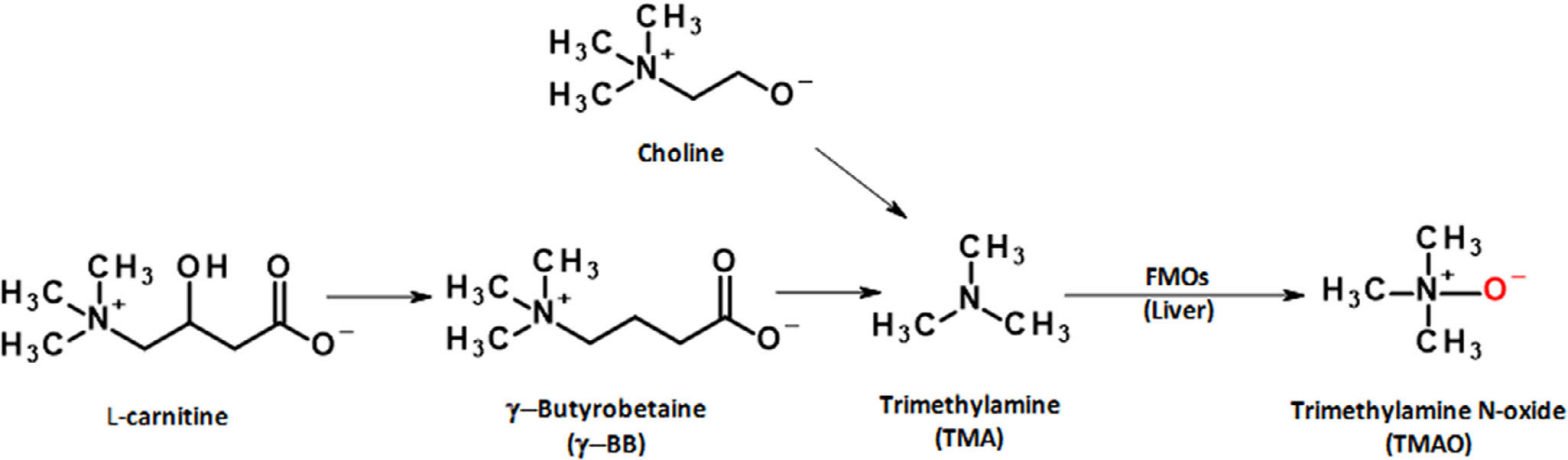
Simplified diagram of choline and l‐carnitine metabolism into trimethylamine N‐oxide (TMAO). l‐Carnitine is first metabolized into its intermediate, γ‐butyrobetaine (γ‐BB), which is then converted to trimethylamine (TMA). Choline is directly converted to TMA. In the liver, TMA is being metabolized into TMAO by flavin‐dependent monooxygenases (FMOs), and TMAO has been reported to be associated with the incidence of various cardiometabolic and other diseases and to cause thrombosis and vascular inflammation.

Trimethylamine *N*‐oxide (TMAO) is generated via a meta‐organismal metabolic route that involves first the gut microbiota‐dependent generation of trimethylamine (TMA) from dietary precursors including l‐carnitine, choline, phosphatidylcholine, and betaine. TMA is subsequently absorbed and oxidized to TMAO by flavin‐dependent monooxygenases (flavin‐containing monooxygenas [FMOs]) in the liver (Figure 1). The TMA precursors are mainly found in animal‐based products such as meat products, dairy, eggs, and fish, although betaine is mainly present in plant‐based products including beets, spinach, and grains [1]. TMAO levels correlate with cardiometabolic diseases and all‐cause mortality risk [2]. A meta‐analysis of 37 studies found that individuals with the highest plasma TMAO levels had a 60% higher risk of all‐cause mortality [2]. Similarly, data from 36 observational studies showed a 74% greater risk of a major adverse cardiovascular event (MACE), while 12 studies reported a 50% greater risk of cardiovascular diseases (CVDs) [2]. In coronary artery disease patients, plasma TMAO levels >6.18 µM were associated with an 88% greater risk of a major cardiovascular event within 3 years of follow‐up compared with subjects who had plasma TMAO levels below <2.43 µM after adjustment for traditional risk factors (e.g., age, sex, blood pressure, diabetes mellitus, cholesterol and triglyceride levels, and smoking status) [3].

Another report, from a human intervention study, in which healthy participants ingested a daily dose of choline for 2 months demonstrated that there was a greater than 10‐fold increase in fasting plasma TMAO levels after 1 month of the intervention, and this elevated level persisted throughout the remainder of the intervention [4]. Arguably the most important finding reported was a significant increase in adenosine diphosphate‐induced platelet aggregation, which significantly correlated to the increase in plasma TMAO levels, suggesting causality [4].

Intervention studies have demonstrated that microbial TMA production can be reduced by broad‐spectrum antibiotics [5] and by the choline analogue 3,3‐dimethyl‐1‐butanol (DMB) [3]. However, the effectiveness of broad‐spectrum antibiotics wears off after 6 months, and long‐term use has a potential to exacerbate the issue of antimicrobial resistance [5]. Other studies have not been able to replicate the effects of DMB [6]. Hence, there is an urgent need for effective and low‐risk treatments that reduce colonic TMA production and consequently reduce the risks associated with high circulating TMAO concentrations.

Dietary components are attractive as potential TMA‐reducing treatments because they are likely to be safe (long prior history of safe consumption), relatively cheap, and are less likely to require formal approvals (such as a favorable outcome of a novel food risk assessment in Europe) before their marketing and use by populations. An important consideration is that the food component(s) will need to reach the microorganisms in the distal gut/colon, where the formation of TMA takes place, to be effective. Dietary polyphenols are poorly absorbed from the small intestine [7]. Consequently, a major fraction of ingested polyphenols reaches the colon where they can interact with the colonic microbiota.

We have previously demonstrated that the in vitro colon model is suitable for studying choline and l‐carnitine fermentation to TMA by the gut microbiome [6]. Few studies have explored the effects of isolated polyphenols on the production of TMA from precursors such as choline and l‐carnitine using in vitro colon models. One study reported that cinnamic acid, ferulic acid, anthocyanin cyanidin‐3‐glucoside, and flavanone hesperidin had the greatest inhibitory effect on TMA production [8]. Others reported that chlorogenic acid and gallic acid could reduce TMA formation from choline in a high‐throughput in vitro human colon model [9].

In vivo evidence is very limited. Reports in rodents found that resveratrol (a stilbene polyphenol), chlorogenic acid, a honeysuckle berry extract, and a blueberry extract reduced plasma and serum TMAO levels in mice and rats [10, 11, 12, 13]. A two‐arm parallel study in 38 healthy men showed that the consumption of black raspberry extract at 30 g/day for 4 weeks could significantly reduce plasma TMAO levels compared to baseline [14]. Similarly, a cross‐over study in 20 healthy men and women showed that the consumption of a grape extract at 600 mg/day for 3 weeks could reduce plasma TMAO levels by 63.6% from baseline [15].

Some of the most poorly absorbed polyphenols are ellagitannins and ellagic acid [16] and anthocyanins [17, 18]; for both only tiny fractions of ingested doses are absorbed from the upper gastrointestinal tract, and the majority reaches the colon. In the colon, polyphenols could interact with the microbiota and in doing so reduce the production of TMA. Pomegranates are good dietary sources of ellagic acid and ellagitannins. Therefore, we aimed to investigate the potential for a polyphenol‐rich pomegranate extract to inhibit the microbial metabolism of the two most common TMA‐precursors, l‐carnitine and choline, using an in vitro model of the human colon.

## 2 Experimental Section

### 2.1 Materials

All water used in this study was 18 MΩ·cm Milli‐Q water, and solvents were of high‐performance liquid chromatography (HPLC) grade, unless otherwise stated. Trichloroacetic acid (TCA), gallic acid, choline chloride‐(trimethyl‐d9), TMA, TMAO, and γ‐butyrobetaine (γ‐BB) (under the commercial name 3‐(carboxypropyl)trimethylammonium chloride) were purchased from Sigma–Aldrich. l‐Carnitine, ellagic acid, acetic acid, ammonium acetate, and all solvents were purchased from Fisher Scientific Limited. Labeled internal standards were purchased from Cambridge Isotope Laboratories (l‐carnitine‐(trimethyl‐d9)), Toronto Research Canada (trimethylamine‐d9 N‐oxide, trimethylamine‐d9 hydrochloride), and Santa Cruz Biotechnology (γ‐Butyrobetaine‐d9). Glacial acetic acid and heptafluorobutyric acid (HFBA) were purchased from Merck. Punicalagin was obtained from BOC Science and punicalin from Apollo Scientific. The Fermac 260 pH control units were from Electrolab and the Stomacher 400 EVO from Seward.

### 2.2 Analysis of the Pomegranate Extract

The pomegranate extract (Dermogranate) was purchased from Medinutrex (Catania, Italy) and has been derived from whole pomegranates. We estimated the quantities of the main pomegranate polyphenols in the extract using LC‐MS/MS. Details of the LC‐MS/MS analysis are provided in the Supporting Information. Briefly, 50 mg of the Dermogranate extract was prepared in 500 µL DMSO and then diluted 20‐fold in 50% v/v aqueous methanol. The extract was filtered before analysis using the Waters TQ Absolute (Wilmslow, UK). The most abundant polyphenols in the pomegranate extract (punicalagin, punicalin, ellagic acid, and gallic acid) were quantified against their matching standards using the retention time (RT) and *m*/*z* [M‐H]^−^. The spectra were inspected using the SYNAPT G2‐Si program.

The polyphenol content was also estimated by an independent lab in Spain (CEBAS‐CSIC in Murcia, Spain, led by Professor Francisco Tomás‐Barberán) using HPLC‐DAD‐MS according to a published method [19]. Briefly, 75 mg of the extract was dissolved in 5 mL of methanol/DMSO/H_2_O (40:40:20 v/v/v) containing 0.1% HCl. The sample was vortexed and filtered through a 0.22 µm PVDF filter before analysis using HPLC‐DAD‐MS.

### 2.3 Study Design

In vitro batch colon model vessels were inoculated with one of four different concentrations of the pomegranate extract (0, 5.7, 11.4, and 22.8 mg/mL), together with 2 mM l‐carnitine or choline and a 1% fecal inoculum. Fecal donations were collected from subjects who consented to participate in the QIB Colon Model study (NCT02653001, ClinicalTrials.gov). Details of the QIB Colon Model study are provided by Day‐Walsh et al. [6]. Briefly, donors included adult men and women, who are based within 10 miles of the Norwich Research Park and have a normal bowel habit, with an average stool type between 3 and 5 on the Bristol Stool Chart, and who did not use antibiotics or probiotics within the 4 weeks before donating. On the day of inoculation of the batch colon model, the donors delivered a fresh fecal sample. Immediately after the fecal sample donation, a 10% slurry of each fecal sample was prepared by diluting the sample 1:10 (w/v) with anaerobic phosphate‐buffered saline (PBS) in a Class II microbiological safety cabinet (MSC). The solution was homogenized using a Stomacher for 2 × 30 s at 230 revolutions per minute (rpm). Each of the donors was allocated a subject code by the colon model manager and only this code was revealed to the researchers in order to maintain donor anonymity. For the same reason, in this study, no subject codes have been used.

The in vitro batch colon models mimic the fermentation conditions in the human colon and the basic conditions were previously reported by Parmanand et al. [20] and Shehata et al. [21]. Briefly, a batch culture medium was prepared containing peptone water, yeast extract, NaCl, K_2_HPO_4_, KH_2_PO_4_, MgSO_4_·7H_2_O, CaCl_2_·6H_2_O, NaHCO_3_, cysteine·HCl, bile salts, Tween80, hemin, and vitamin K_1_. After autoclaving, a concentrated stock of sterile filtered D‐glucose was added to the media, reaching a final concentration of 1%. Glass vessels of 300 mL volume were sealed and autoclaved. The vessels were connected to a circulating water bath (Grant Instruments Ltd, Shepreth, UK), such that the temperature of the vessels was kept at 37°C. The pH of vessels was maintained between 6.6 and 7.1 using a pH controller, which was connected to NaOH (0.5 M) and HCL (0.5 M) pumps to maintain the pH within the preset range. Furthermore, anaerobic conditions were maintained through continuously sparging the media with oxygen‐free nitrogen.

Before fecal inoculation, 100 mL of autoclaved batch culture medium was added to each vessel, and the oxygen was removed from the vessels overnight, using a continuous oxygen‐free nitrogen flow. The vessels were then inoculated with 11 mL fresh fecal inoculum (1% final concentration of fecal matter), together with the pomegranate extract and 2 mM l‐carnitine or choline. Choice of substrate concentrations has been clarified in the Supporting Information. Samples for microbial metabolite analyses were taken at baseline and at multiple time points thereafter. Samples were stored at −80°C directly after collection until LC‐MS/MS analysis.

In total, 9 donors made 17 donations. A separate donation was used for each experiment. Two sets of experiments were conducted, the first testing the effect of a single pomegranate extract dose (5.7 mg) over 24 h. Following these experiments, we sought to confirm the effect observed using a second set of experiments testing a range of doses (5.7, 11.4, 22.8 mg) over 48 h.

### 2.4 Dosage Information

The pomegranate extract doses (5.7, 11.4, and 22.8 mg/mL) were chosen based on a total polyphenol content of 20%, as stated by the manufacturer (Medinutrex). Hence, the increasing doses correspond to 1.14, 2.28, and 4.56 mg/mL of polyphenols, respectively. The human colon has been estimated to hold an average volume of 561 mL (including the ascending, transverse, and descending colon) [22]. This equates to 639.5–2558 mg of polyphenols in the human colon, which reflects the range of doses used in previously reported human studies investigating pomegranate extracts and juices with quantities that range between 435 and 2660 mg of polyphenols (gallic acid equivalents [GAEs]) [23, 24, 25, 26]. The highest dose used in the experiments described here can be achieved by the consumption of 500 mL of pomegranate juice, containing approximately 2200–2560 mg of polyphenols [25, 27], with the intermediate and low doses corresponding to approximately 250 and 125 mL of juice, respectively.

### 2.5 Sample Preparation for LC‐MS/MS

LC‐MS/MS was used to quantify the γ‐BB, l‐carnitine, and TMA present in the samples collected at each time point using a previously reported method (Day‐Walsh et al.). Briefly, samples were mixed with TCA and a mixture of d9‐labeled reference standards (choline‐d9, l‐carnitine‐d9, γ‐BB‐d9, TMA‐d9, and TMAO‐d9 prepared in 0.2 M aqueous acetic acid), centrifuged, and the final samples prepared by dilution 1 in 20 with water. Alongside, serial dilutions containing external standards (choline, l‐carnitine, γ‐BB, TMA, and TMAO) and the same amount of each d9‐labeled reference standard as added to samples were prepared in the matrix containing 1% fecal inoculum and colon model media. All vials were capped (Agilent Technologies) and stored at −20°C until analysis.

### 2.6 Metabolite Quantification

A detailed description of the metabolite quantification using LC‐MS/MS is described elsewhere [6]. The obtained peak areas of the samples were divided by the peak area of the internal standard to obtain peak area ratios. Absolute concentrations were calculated from the peak area ratios using the standard curves obtained for the external standards over a range of known concentrations. Any changes in the volume of the batch model, caused by the addition of acid/base to maintain the pH, were adjusted for in the calculations.

### 2.7 Microbial Plating to Count CFUs

Six in vitro batch colon models were set up, of which three contained the pomegranate extract at 22.8 mg/mL and three were used as controls. All models were inoculated with 1% fecal inoculum of an individual donor. Samples for microbial plating were taken at 0, 6, and 24 h. Plates were prepared using autoclaved colon model media with 1% agar and were made anaerobic overnight in an anaerobic cabinet. After samples were collected from the colon model vessels, serial dilutions were prepared in the anaerobic cabinet, ranging from 10^−1^ to 10^−7^. From the serial dilutions, 5 µL samples were transferred onto agar plates in triplicate. After 24 h the colony‐forming units (CFUs) were counted.

### 2.8 Statistical Analyses

Average trajectories of metabolites over time were calculated by first taking mean averages of replicates within donors, the displayed means and standard errors were then calculated by comparing these averages across donors. One data point that was an obvious measurement error was removed before graphing and analysis, and the data for one control was missing due to an error in the experiment.

The within‐donor effect of pomegranate on concentrations of substrates and metabolites was then estimated using linear mixed models, with treatment as fixed effects and random intercepts of donors. Independent models were estimated for each outcome at each time point. Although *p* values from these models give some indication of average differences between treatments, the data suggest that the rate of conversion and the effects of pomegranate are likely to depend on the donor. There was not enough data to model these donor‐specific effects using, for instance, random slope models, nor to model nonlinear dose–response relationships, and so *p* values based on random intercept models should be treated with some caution. Dose–response was tested by replacing doses with equally spaced numerical codes and estimating the linear effect of increasing dose.

Logistic growth curves were also used for modeling trajectories of metabolites over time, with subsequent testing of model parameters (inflection point and growth rate) across conditions using linear mixed models, but there was not enough data for good quality curve fits in many cases. For logistic curve fits, observed γ‐BB and TMA concentrations were replaced by their cumulative maximums to closely reflect their total production. In two cases, for γ‐BB production from l‐carnitine with the highest dose of pomegranate (Donors 1 and 2), the production was delayed such that the upper asymptote of the curve could not be estimated, and so an upper bound was set manually. Changing this bound did not alter the results of the analysis.

All statistical analysis was conducted using R, mixed modeling used lme4 [28], lmerTest [29], and broom.mixed [30] packages. Nonlinear modeling was conducted using minpack.LM [31].

## 3 Results

### 3.1 Characterization of the Pomegranate Extract

Several peaks which were identified as polyphenols appeared on the chromatogram between RTs 2.18 and 7.31 min, with gallic acid appearing first and ellagic acid last (Table 1). Two peaks were detected for punicalagin (Figure S1), which likely corresponds with the alpha and beta isomers. For punicalin, a split peak was measured (Figure S2), which likely reflects the presence of a mixture of the alpha and beta isomers of punicalin.

**TABLE 1:** Quantification of the main polyphenols in the Dermogranate extract.

Compound	RT (min)	*m*/*z* [M–H]^−^	Quantity (µg/mg extract)
Gallic acid	2.18	169	2.7
Punicalin a + b	3.37	781	51.5
Punicalagin a + b	4.81/5.31	1083	50.8
Ellagic acid	7.31	301	11.3

*Note*: Triplicate samples of the Dermogranate extract were dissolved in 5% dimethyl sulfoxide (DMSO) in aqueous methanol (1:1, v/v) and analyzed using LC‐MS/MS as described in Section 2.

Punicalin made up the largest proportion of polyphenols in the pomegranate extract, contributing to 5.15% of the total weight of the extract, followed by punicalagin with 5.08% of the total weight, and ellagic acid with 1.13% of the total weight (Table 1). Gallic acid makes up only a small proportion of the extract. Based on the sum of the contents of the polyphenols we measured, they accounted for 11.63% of the extract on a mass basis. Broadly similar results were obtained when the extract was analyzed by an independent lab (data not shown).

### 3.2 The Pomegranate Extract Delayed and Slowed the Microbial Conversion of Both Choline and l‐Carnitine to TMA

**FIGURE 2:**
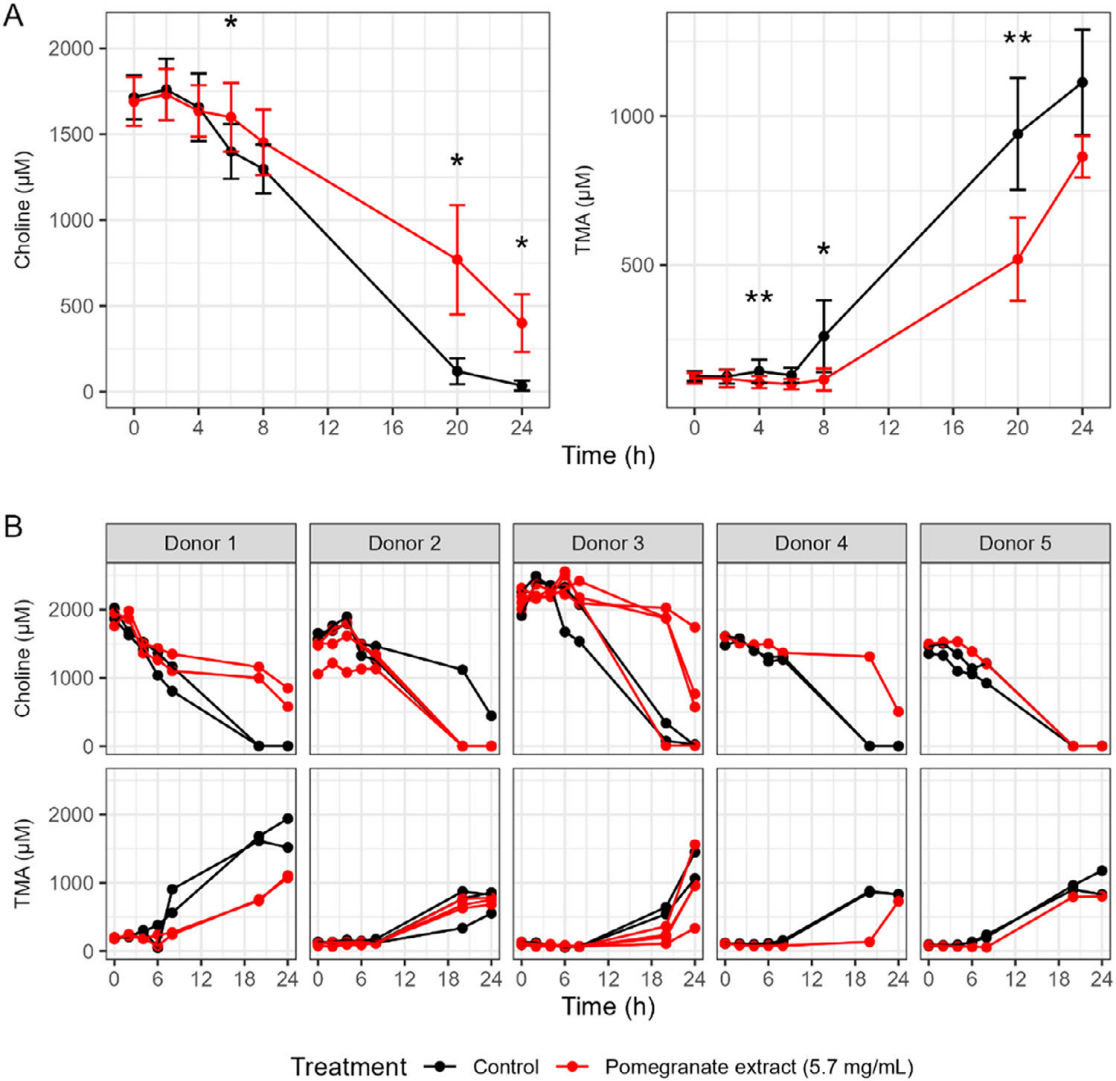
Choline and trimethylamine (TMA) concentrations in in vitro colon models treated with pomegranate extract. (A) Average concentrations of choline and TMA across donors and replicates, comparing vessels treated with pomegranate extract (5.7 mg/mL) to control vessels at each time point over 24 h. Data are shown as mean ± standard deviation (SD) across all donors and *p < 0.05 and **p < 0.01. (B) Individual vessel trajectories stratified by donor. In vitro batch colon models were inoculated with a 1% fecal inoculum from a healthy donor. Vessels contained 2 mM choline, with or without pomegranate extract. Samples were collected at baseline and at multiple time points over 24 h, then stored at −80°C until analysis. Choline and TMA concentrations were quantified using LC‐MS/MS with isotope‐labeled internal standards.

**FIGURE 3:**
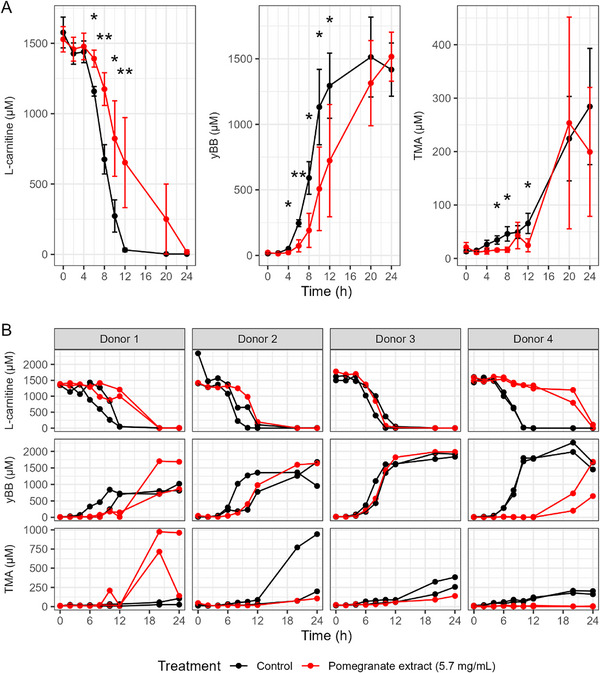
l‐Carnitine, total γ‐butyrobetaine (γ‐BB), and trimethylamine (TMA) concentrations in in vitro colon models treated with pomegranate extract. (A) Average concentrations of l‐carnitine, total γ‐BB, and TMA across donors and replicates, comparing vessels treated with pomegranate extract (5.7 mg/mL) to control vessels at each time point over 24 h. Data are shown as mean ± standard deviation (SD) across all donors and *p < 0.05 and **p < 0.01. (B) Individual vessel trajectories stratified by donor. In vitro batch colon models were inoculated with a 1% fecal inoculum from a healthy donor. Vessels contained 2 mM l‐carnitine, with or without pomegranate extract. Samples were collected at baseline and multiple time points up to 48 h, then stored at −80°C until analysis. Metabolite concentrations were quantified using LC‐MS/MS with isotope‐labeled internal standards.

Figures 2 and 3 show the results from initial experiments monitoring the effect of 5.7 mg/mL pomegranate extract on the production of TMA from choline and the production of TMA and γ‐BB from l‐carnitine over 24 h. The average concentrations across donors and replicates of the substrate and metabolites are shown, with *p* values comparing the averages of treated versus control vessels at each time point (Figures 2A and 3A). The trajectories from individual vessels, stratified by donor, are shown in Figures 2B and 3B.

The pomegranate extract at 5.7 mg/mL prevented the depletion of choline and the corresponding production of TMA, with some significant differences in concentrations at different time points across the experiment, but the trajectories and effects appear to vary by donor (Figure 2B).

Similarly, the pomegranate extract at 5.7 mg/mL delayed the conversion of l‐carnitine into γ‐BB and TMA, with differences in averages seen in all products between 6 and 12 h (Figure 3), but as with choline, there were inconsistent patterns observed across donors (Figure 2B).

Since these data suggested that the pomegranate extract might be effective in reducing the metabolism of choline and l‐carnitine to TMA and γ‐BB, a second set of experiments was conducted with a range of doses (0, 5.7, 11.4, and 22.8 mg/mL) to explore potential dose–response effects. Additionally, the time of the experiment was increased to 48 h such that the full metabolic trajectories could be captured, in particular, the trajectory of TMA production.

### 3.3 Dose‐Dependent Inhibition of l‐Carnitine Conversion to TMA by the Pomegranate Extract

**FIGURE 4:**
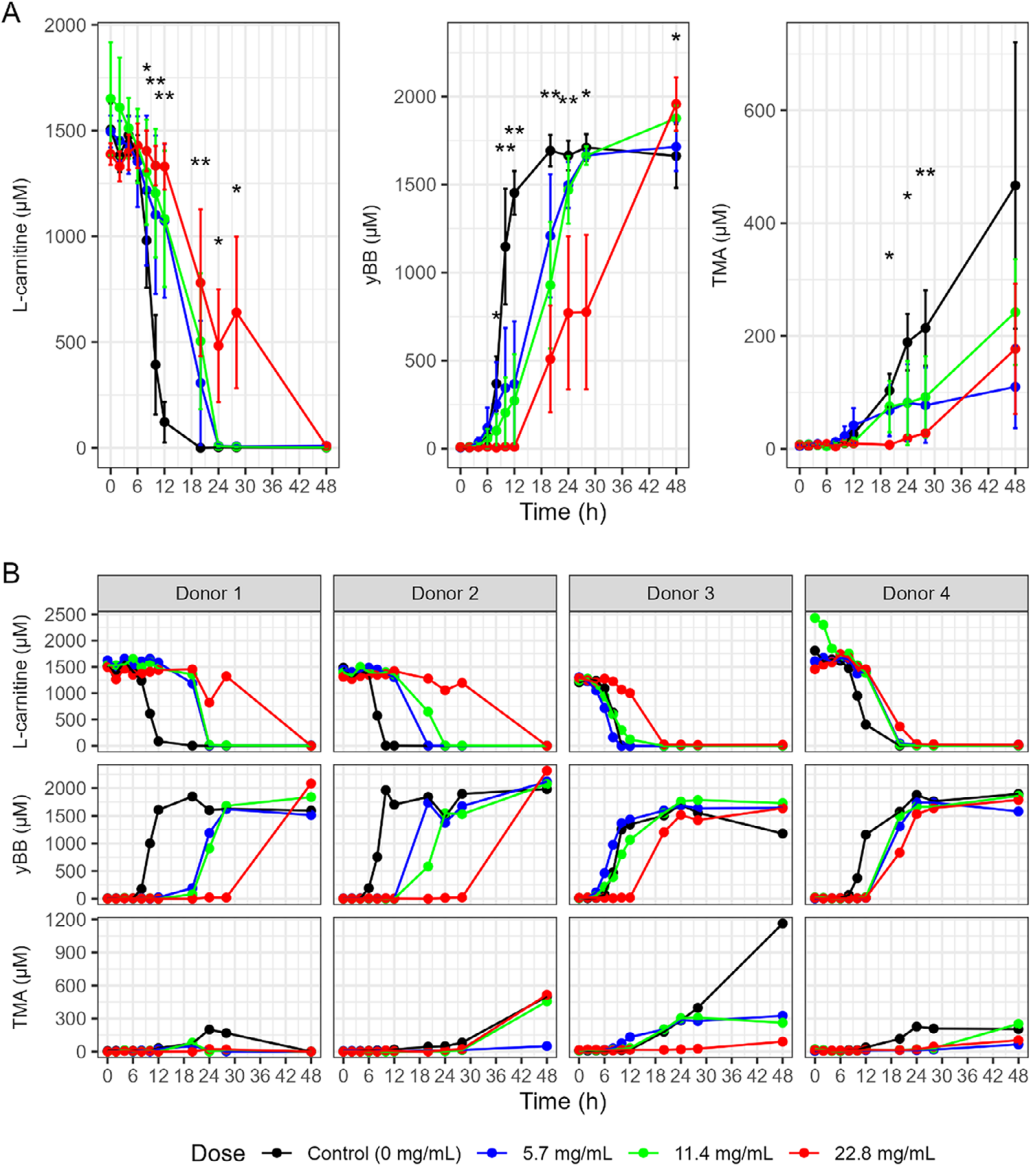
Dose–response effect of pomegranate extract on l‐carnitine metabolism in in vitro colon models. (A) Average concentrations of total γ‐butyrobetaine (γ‐BB) and trimethylamine (TMA) over 48 h across all donors, comparing vessels treated with increasing concentrations of pomegranate extract (5.7, 11.4, and 22.8 mg/mL). Data are shown as mean ± standard deviation (SD) across all donors and *p < 0.05, **p < 0.01. (B) Individual vessel trajectories stratified by donor. In vitro batch colon models were inoculated with a 1% fecal inoculum from a healthy donor. Vessels contained 2 mM l‐carnitine, with or without pomegranate extract. Samples were collected at baseline and multiple time points up to 48 h, then stored at −80°C until analysis. Metabolite concentrations were quantified using LC‐MS/MS with isotope‐labeled internal standards.

The data provide strong evidence that pomegranate extract dose‐dependently delays the conversion of l‐carnitine to γ‐BB (Figure 4). This appeared to be largely consistent across donors (Figure 4B), with statistically significant average dose–response effects in all curves at different time points. The effect on the production of TMA from l‐carnitine was less pronounced, owing to very variable production across donor samples.

### 3.4 Dose‐Dependent Inhibition of Choline Conversion to TMA by the Pomegranate Extract

**FIGURE 5:**
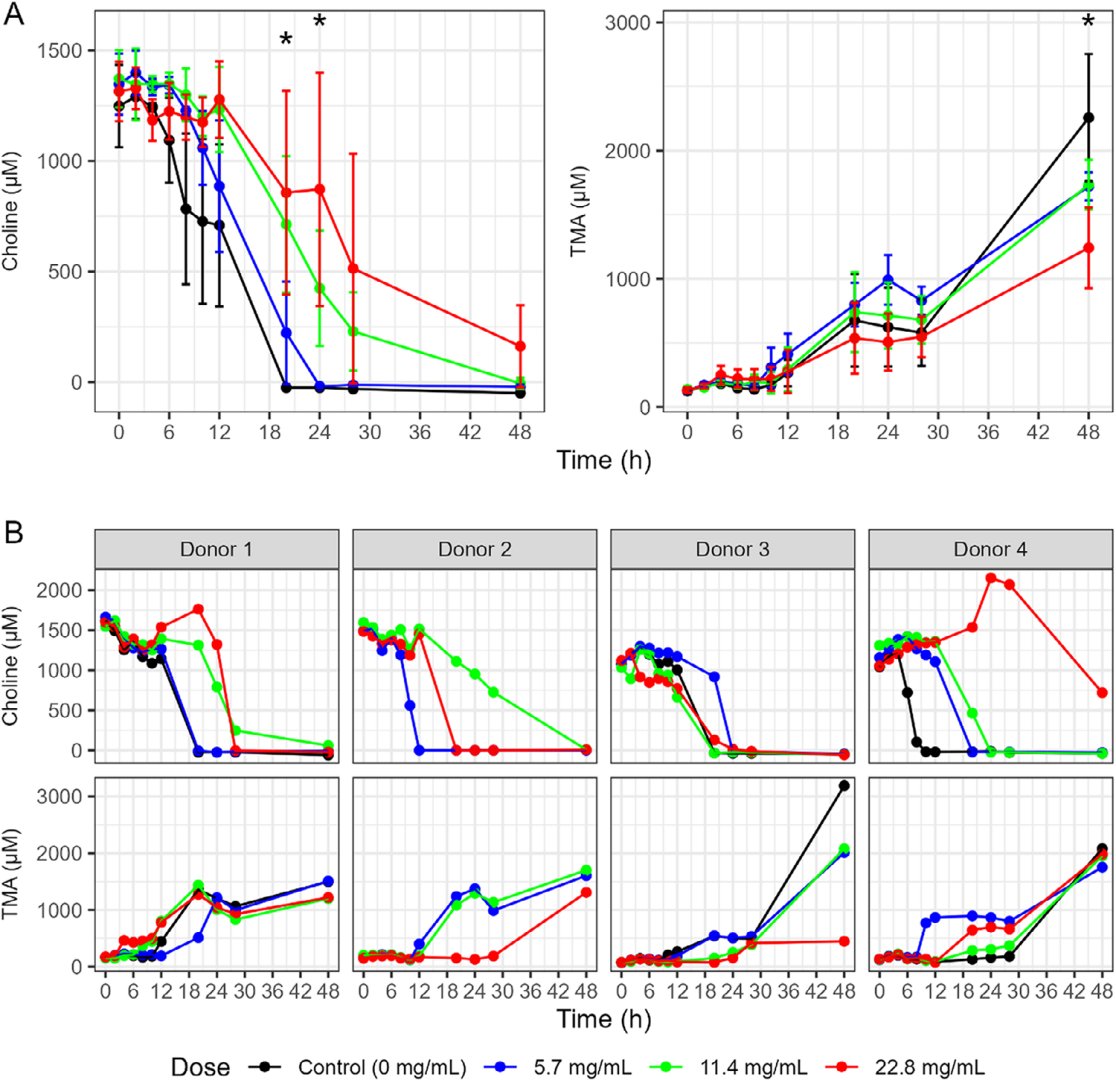
Dose–response effect of pomegranate extract on choline metabolism in in vitro colon models. (A) Average concentrations of trimethylamine (TMA) over 48 h across all donors, comparing vessels treated with increasing concentrations of pomegranate extract (5.7, 11.4, and 22.8 mg/mL). Data are shown as mean ± standard deviation (SD) across all donors and *p < 0.05. (B) Individual vessel trajectories stratified by donor. In vitro batch colon models were inoculated with a 1% fecal inoculum from a healthy donor. Vessels contained 2 mM choline, with or without pomegranate extract. Samples were collected at baseline and multiple time points up to 48 h, then stored at −80°C until analysis. Metabolite concentrations were quantified using LC‐MS/MS with isotope‐labeled internal standards.

On the other hand, there was no consistent effect of the different pomegranate extract doses on the metabolism of choline to TMA (Figure 5). Although on average there was some evidence of lower choline metabolism and higher TMA production at the midpoint of the experiment with higher doses of pomegranate (Figure 5A), this seemed extremely variable across donor samples, and no consistent pattern was observed (Figure 5B). Notably, there was an elevation of the choline concentration after 12 h of incubation for Donors 1 and 4 (Figure 5B), which could be explained by the release of choline from other precursors found in the fecal material, such as phosphatidylcholine (via microbial phospholipase activity).

### 3.5 The Effect of the Pomegranate Extract on l‐Carnitine Metabolism Using Logistic Growth and Decay Models

**FIGURE 6:**
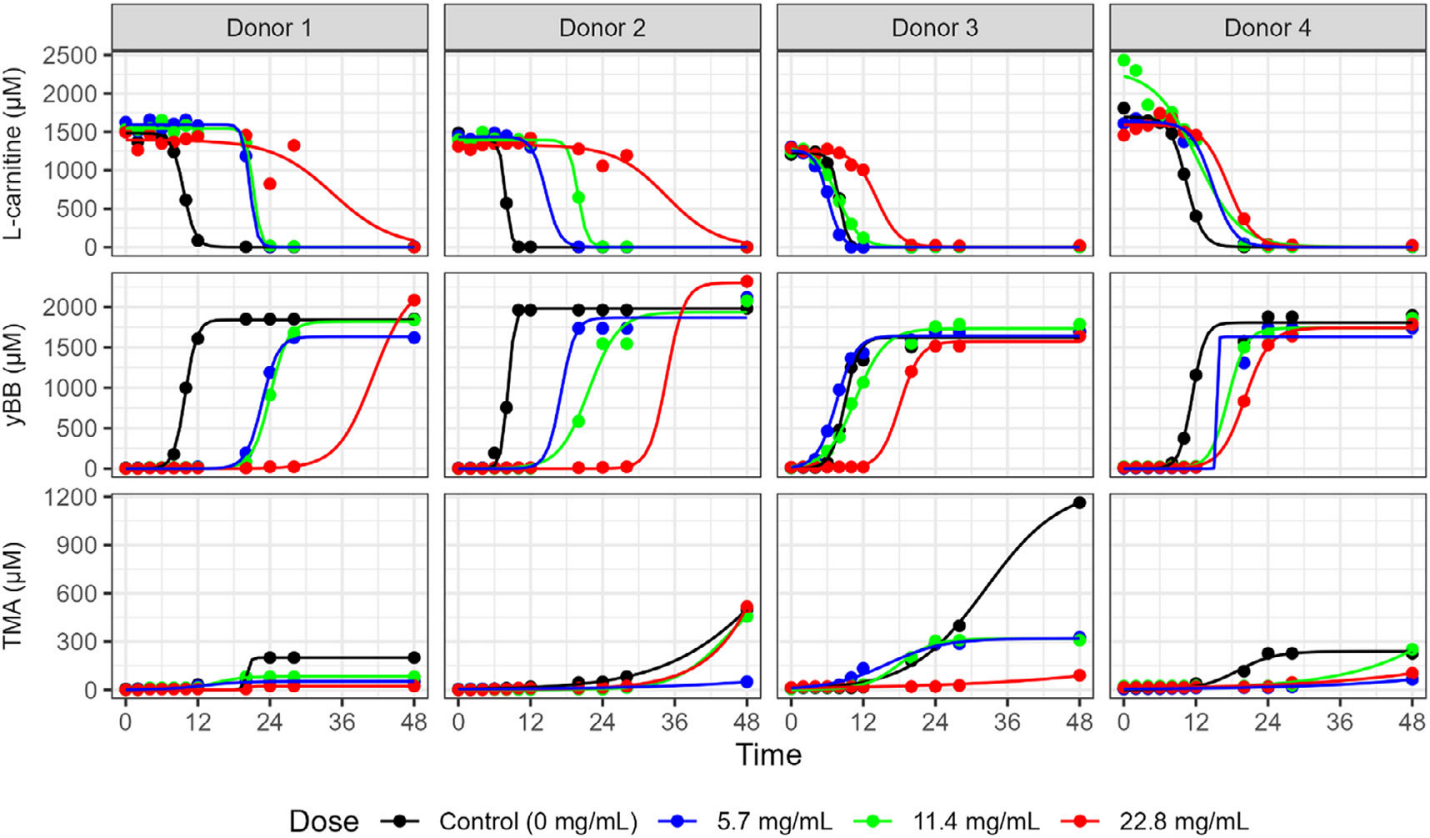
Logistic growth and decay modeling of pomegranate extract's dose–response effect on l‐carnitine metabolism. Modeled trajectories of γ‐butyrobetaine (γ‐BB) and trimethylamine (TMA) concentrations over 48 h in in vitro batch colon models, stratified by donor. Models incorporate a logistic growth and decay function to describe the dose–response effect of pomegranate extract (5.7, 11.4, and 22.8 mg/mL) on metabolite production. Vessels were inoculated with a 1% fecal inoculum from a healthy donor. Experimental data were fitted to nonlinear models, with curve parameters detailed in Figure S3.

As a secondary analysis, we also applied nonlinear regression to model the metabolism of each metabolite using logistic growth and decay models. However, in many cases, there was insufficient data with which to reliably estimate a curve, limiting the comparisons that could be made. Figure 6 shows these curves for the metabolism of l‐carnitine to γ‐BB and TMA; comparing the curve parameters across conditions generates similar results as the primary analysis, in which each time point was individually compared.

For l‐carnitine metabolism and γ‐BB production, there were significant dose–response effects of the pomegranate extract on both the time of the inflection point of the curve, with increasing levels of pomegranate leading to a longer delay, and on the scale of the curve, with increasing pomegranate leading to a slower rate of metabolism or growth (Supplementary Table S1, Figure S3). The highest dose (22.8 mg/mL) delayed the average midpoint of the l‐carnitine metabolism curve by 16 h (95% CI = 8.4‐24; *p* = 0.001) compared to the control condition, while the highest dose delayed γ‐BB production by 19 h (95% CI = 11‐27; *p* < 0.01). For TMA production, these differences were not evident, but production over the course of the experiment did not plateau in many cases, meaning that growth curves were difficult to estimate.

### 3.6 Impact of the Pomegranate Extract on Viable Bacterial Numbers

**FIGURE 7:**
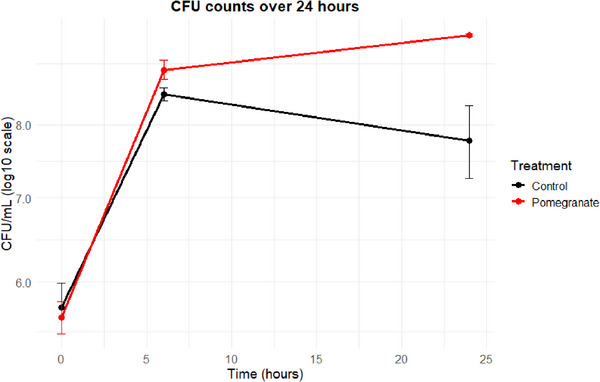
Bacterial growth in in vitro colon models measured as colony‐forming units (CFUs) per mL over 24 h. Six in vitro batch colon models were set up, three containing pomegranate extract (22.8 mg/mL) and three serving as controls. All models were inoculated with a 1% fecal inoculum from a single donor. Samples for microbial plating were collected at 0, 6, and 24 h. Serial dilutions (10^−1^ to 10^−7^) were prepared in an anaerobic cabinet, and 5 µL of each dilution was plated in triplicate onto anaerobic agar plates. CFUs were counted after 24 h of incubation. Data are presented as mean ± standard deviation (SD) from three biological replicates (*n* = 3).

It should be considered that the pomegranate extract has antibacterial effects, and therefore the observed reductions in choline and l‐carnitine metabolism could be due to reductions in the growth and/or viability of the fecal microorganisms. Therefore, viable bacterial numbers were quantified (Figure 7) in three replicates from a single donor. For both the control and the pomegranate‐treated conditions, viable bacterial numbers increased rapidly between 0 and 6 h to 2.94 × 10^8^ and 7.34 × 10^8^, respectively. Between 6 and 24 h, the number of CFUs increased in the pomegranate‐treated condition but decreased slightly in the control condition (Figure 7). These data show that the pomegranate extract treatment did not reduce total viable bacterial numbers.

## 4 Discussion

In this study, we investigated the potential of a pomegranate extract to inhibit the production of TMA from choline and l‐carnitine. The pomegranate extract showed some inhibitory effects on the metabolism of choline to TMA, although there was not a consistent dose–dependent effect across donors. A more consistent dose‐dependent inhibitory effect was observed on the microbial metabolism of l‐carnitine to γ‐BB and TMA. The highest pomegranate extract dose tested (22.8 mg/mL) significantly delayed the metabolic conversion of l‐carnitine. Logistic growth and decay models supported the observed dose‐dependent effects on l‐carnitine metabolism, confirming delays in the inflection points for both l‐carnitine and γ‐BB curves with increasing concentrations of the pomegranate extract. At the highest dose, the time to reach half‐maximum metabolite concentrations (half‐maximal inhibitory concentration [IC_50_]) was significantly prolonged for l‐carnitine as well as γ‐BB, with a 16‐h delay in l‐carnitine depletion and a 19‐h delay in γ‐BB production compared to the control condition.

There are three potential mechanisms through which the pomegranate extract causes the effects reported here.

First, the inhibition of l‐carnitine and choline metabolism may have been a consequence of the pomegranate polyphenols inhibiting the growth or viability of microorganisms that are involved in the metabolism of l‐carnitine and/or choline. Polyphenols have both antimicrobial effects and growth stimulating effects on microorganisms [32]. Since we observed an overall increase in total viable bacteria when the in vitro models were treated with the pomegranate extract, the extract is not acting as a general antibacterial, but it may be affecting the abundance of specific microorganisms responsible for the metabolism of l‐carnitine, γ‐BB, and choline, while promoting overall microbial growth. The effect induced by polyphenols, that is, growth stimulating or antimicrobial, depends on the type of polyphenol as well as the type of microorganism. Pomegranate polyphenols have strong antimicrobial effects on gut microbial species, including *Escherichia coli*, *Proteus* sp., *Salmonella typhimurium*, and other *Salmonella* species [33, 34]. These species are also known to contain *cai* genes, as well as the *cutC* gene, which are involved in the anaerobic metabolism of l‐carnitine to γ‐BB and choline to TMA, respectively [35, 36]. At the same time, pomegranate polyphenols are known to stimulate the growth of probiotic species such as *Bifidobacterium breve, B. infantis*, other *Bifidobacterium* species, *Lactobacillus* sp., and *Akkermancia muciniphila* [34, 37]. Growth promoting effects can be the result of the enzymatic release of glucose and free gallic and ellagic acid from the polyphenols [32, 38].

Second, the pomegranate extract may affect the expression of the genes responsible for the metabolism of l‐carnitine, γ‐BB, and choline, which can occur at the level of transcription and/or at the level of translation. Polyphenols can interact with proteins that are responsible for the transcription as well as the expression of genes that are associated with metabolism [39]. This mainly happens through the production of metabolites during the fermentation of polyphenols [40]. Due to their antioxidant properties, polyphenols may interact with the transcription and expression of genes associated with metabolism [41]. Since the microbial metabolism of l‐carnitine and choline into TMA depends on metabolic enzymes (such as those encoded by the *cai* and *cut* genes), the redox environment shaped by the pomegranate polyphenols could influence the gene expression of these microbial enzymes.

Third, the pomegranate extract may inhibit the function(s) of the proteins involved in the metabolism of l‐carnitine, γ‐BB, and choline, for example, the antiporter proteins and/or the enzymes. Several polyphenols have been shown to form a complex with the extracellular component of transmembrane proteins (e.g., porins) from microorganisms, thereby modulating the microbial membrane permeability [42, 43]. By altering the microbial membrane permeability, polyphenols may affect the availability of l‐carnitine, γ‐BB, and choline to microbial enzymes, potentially affecting their conversion into TMA. Alternatively, polyphenols may be taken up by bacterial cells and affect the activity of their intracellular catalytic machinery.

There are a few reports that provide evidence that dietary polyphenols and polyphenol‐rich foods can reduce TMA production and TMAO levels [44]. Several of these reports describe experiments with isolated polyphenols, others with polyphenol‐rich extracts, and some with much more complex polyphenol‐rich foods. It is important to recognize the strengths and weaknesses of the different types of polyphenol treatments. For example, whole (complex) foods might seem to be a sensible way of investigating the effects of food polyphenols because it retains the food context (matrix), but (i) it is very difficult to generate a true control intervention (i.e., the same food, but lacking the polyphenols) and (ii) the macronutrients present will substantially affect the gut microbiota and probably also the chemical properties of the colonic environment. An example of this is reported by Bresciani et al. (2018), who investigated the effects of orange juice, grapefruit juice, and pomegranate juice on the production of TMA from l‐carnitine [8]. The authors noted that the greatest effects observed were caused by the sugars in the juices, which was probably attributed to the pH‐lowering effects. For this reason, it is likely that in vitro colon model experiments conducted with isolated polyphenols or purified polyphenol‐rich extracts are more reliable than those conducted with macronutrient‐rich foods. Therefore, we used a pomegranate extract and monitored the pH of the in vitro colon models. Since the pH was kept stable, the observed TMA reduction cannot be attributed to the pH‐lowering effects of the extract.

To the best of our knowledge, the pomegranate extract is the most effective nutraceutical to reduce the production of γ‐BB and TMA from l‐carnitine by human fecal microbiota reported to date, although it has only been demonstrated in an in vitro model of the human colon. In a study by Iglesias‐Carres et al. (2021) in which an in vitro model of the human colon was also used, it was reported that chlorogenic and gallic acid could reduce the microbial metabolism of choline to TMA [9]. This study adds new findings to the small body of evidence for constituents that can reduce TMA production from choline, with the pomegranate extract being an effective inhibitor. However, we do not know which constituent(s) of the extract are responsible for the observed inhibitory effects. The effects may be attributed to the main polyphenols in the pomegranate extract (punicalagin, punicalin, ellagic acid, and gallic acid) or to their derivatives, urolithins. Within the human gut, punicalagin is converted to punicalin, and then to ellagic acid [45, 46]. Within the colon, ellagic acid is converted to various urolithins [45, 46]. Urolithins have been attributed to gut microbiota modulating effects, being prebiotic to some microbial species and inhibitory to others [37].

A key limitation of this study is that the in vitro colon models simulate colonic fermentation but do not account for digestion in other parts of the gastrointestinal tract. For example, the pomegranate extract may undergo metabolism in the small intestine, altering the compounds that reach the colon. Published reports have shown that ellagitannins, such as punicalagin from pomegranate, are partly broken down into ellagic acid before reaching the colon [46], meaning that the mix of ellagic acid and ellagitannins used to treat the colon models is not likely to be the same as the colon would be exposed to in vivo. Additionally, substantial interindividual variability exists between donors, which reduces the chances of detecting significant differences caused by treatments. Given these limitations, further studies in human subjects are essential to confirm these findings.

The data presented here highlight the potential of a pomegranate extract to selectively modulate gut microbial pathways associated with TMA formation, a precursor to proatherogenic TMAO, suggesting their use as dietary interventions for cardiometabolic health. Future studies are warranted to investigate the individual pomegranate polyphenols (punicalagin, punicalin, ellagic acid, and gallic acid) and derivatives (urolithins) for their TMA‐inhibiting effects. In addition, the polyphenol‐rich pomegranate extract should be tested for its effectiveness in reducing plasma TMAO responses to an l‐carnitine dose in a healthy study population and subsequently those at a high risk of cardiometabolic diseases.

## 5 Concluding Remarks

In this study, we have demonstrated that a pomegranate extract rich in the polyphenols punicalagin, punicalin, and ellagic acid significantly modulated the microbial metabolism of l‐carnitine and choline in vitro, with a dose‐dependent inhibition of γ‐BB and TMA production from l‐carnitine. The observed metabolic shifts were not due to a general bactericidal effect since pomegranate‐treated fermentation vessels contained greater numbers of viable bacteria compared to controls.

## Conflicts of Interest

The authors declare no conflicts of interest.

## Supporting information




**Supporting file**: mnfr70166‐sup‐0001‐SuppMat.docx

## Data Availability

The data that support the findings of this study are openly available in Zenodo at http://doi.org/10.5281/zenodo.15039153.
