# Supplementary material for: A Pomegranate Polyphenol Extract Suppresses the Microbial Production of Proatherogenic Trimethylamine (TMA) in an In Vitro Human Colon Model
Source: Mol Nutr Food Res. 2025 Jun 29;69(20):e70166. doi: 10.1002/mnfr.70166 (PMC12538525; doi:10.1002/mnfr.70166)
Supplement: Supplementary file 1 — Supporting file: mnfr70166‐sup‐0001‐SuppMat.docx [file MNFR-69-e70166-s001.docx]

**Supplementary Information**

**A pomegranate polyphenol extract suppresses the microbial production of proatherogenic trimethylamine (TMA) in an *in vitro* human colon model**

Julia E Haarhuis^1 *^, Priscilla Day-Walsh^1,2,3 *^, Emad Shehata^1,4^, George M Savva^1^, Barbora Peck^1^, Mark Philo^1^, Paul A Kroon^1^

^1^Quadram Institute Bioscience, Norwich Research Park, Norwich, NR4 7UQ, UK.

^2^Department of Obstetrics and Gynaecology, University of Cambridge, The Rosie Hospital, Robinson Way, Cambridge CB2 0SW, UK.

^3^Centre for Trophoblast Research (CTR), Department of Physiology, Development and Neuroscience, University of Cambridge, Cambridge, CB2 3EG, UK.

^4^Food Industries and Nutrition Research Institute, National Research Centre, 33 El Buhouth St., Dokki 12622, Cairo, Egypt.

^*^ Shared first authors.


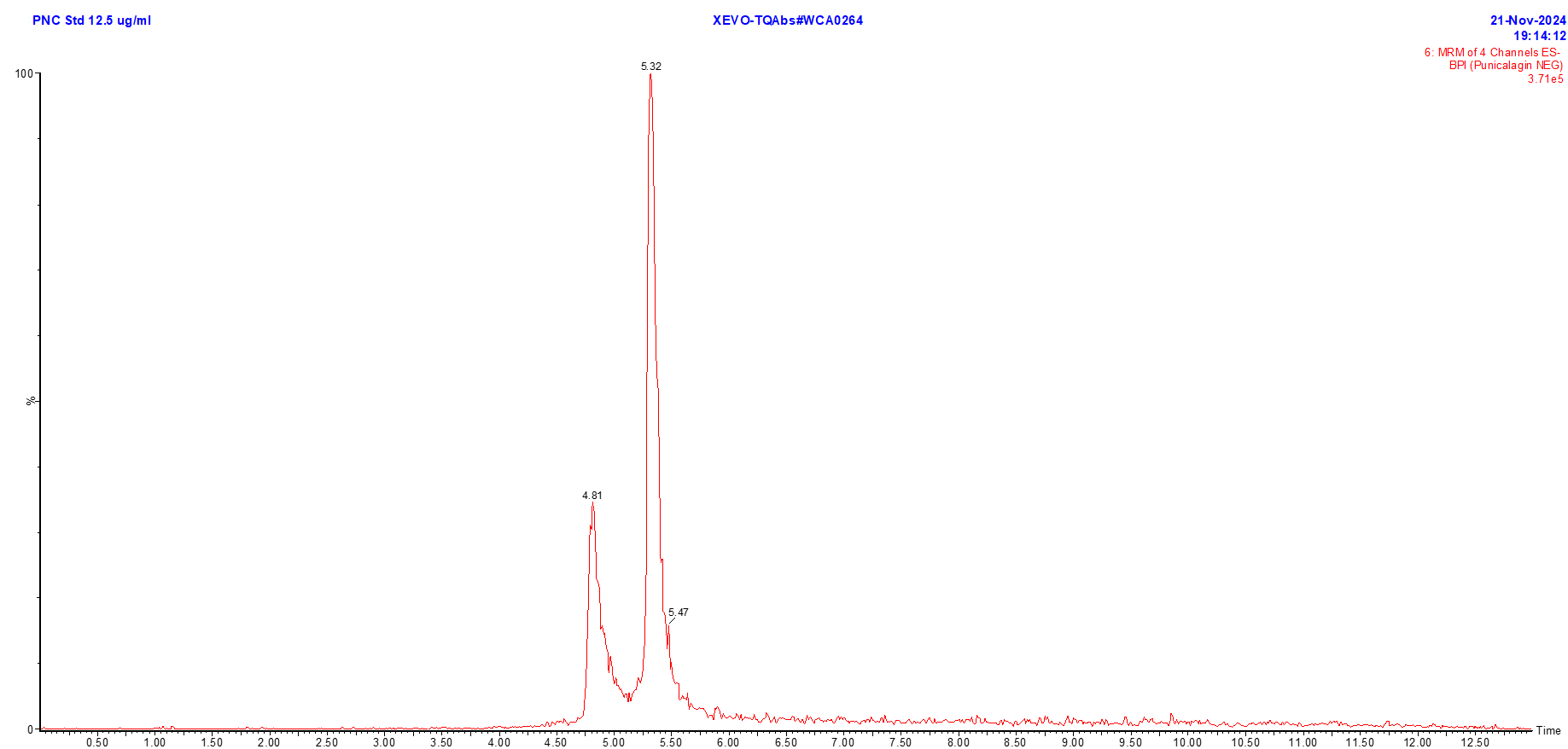


**Supplementary Figure S1. LC-MS total ion current (TIC) of a punicalagin standard at 12.5 µg/mL.** Punicalagin was dissolved in 50% aqueous methanol, filtered, and analyzed using LC-MS/MS. Two peaks were identified, corresponding to the alpha and beta isomers of punicalagin. A Waters TQ Absolute (Wilmslow, UK) system was used with a mobile phase of 0.1% formic acid in distilled water (eluent A) and 0.1% formic acid in acetonitrile (eluent B). 1 µL of the punicalagin standard was run through a Luna Omega Polar C18 100A column (100 x 2.1 mm; particle size 1.6 µm) at 35°C and a flow rate of 400 µL/min. The gradient was 3% eluent B for 5 minutes, after which eluent B was increased to 20% for 4 minutes, and then further increased to 50% for 2 minutes, lastly eluent B was increased to 95% for 1 minute before it was re-equilibrated to 3% for 2 minutes.


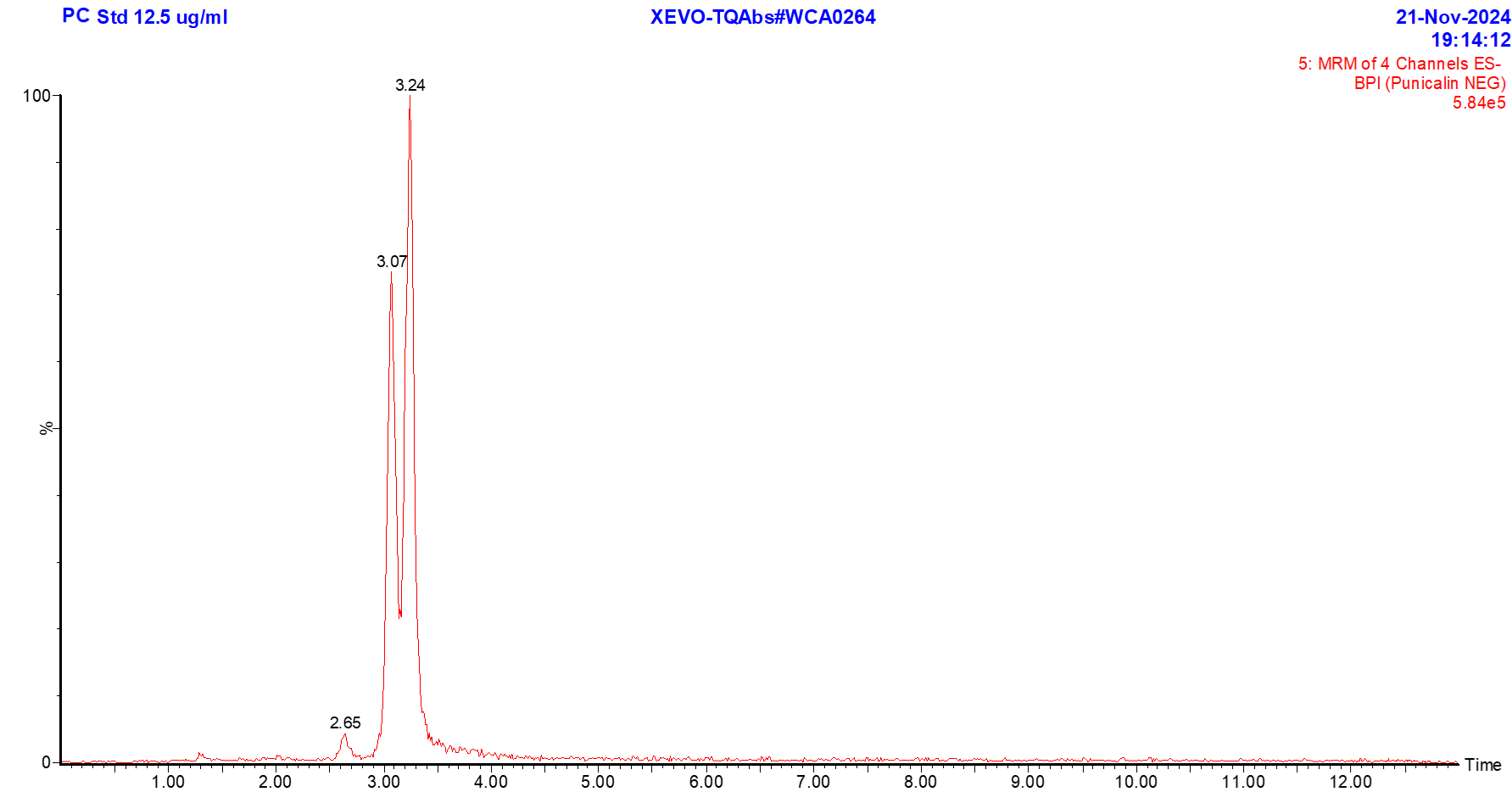


**Supplementary Figure S2. LC-MS total ion current (TIC) of a punicalin standard at 12.5 µg/mL.** Punicalin was dissolved in 50% aqueous methanol, filtered, and analyzed using LC-MS/MS. A split peak was identified, indicating the presence of a mixture of alpha and beta isomers. A Waters TQ Absolute (Wilmslow, UK) system was used with a mobile phase of 0.1% formic acid in distilled water (eluent A) and 0.1% formic acid in acetonitrile (eluent B). 1 µL of the punicalin standard was run through a Luna Omega Polar C18 100A column (100 x 2.1 mm; particle size 1.6 µm) at 35°C and a flow rate of 400 µL/min. The gradient was 3% eluent B for 5 minutes, after which eluent B was increased to 20% for 4 minutes, and then further increased to 50% for 2 minutes, lastly eluent B was increased to 95% for 1 minute before it was re-equilibrated to 3% for 2 minutes.


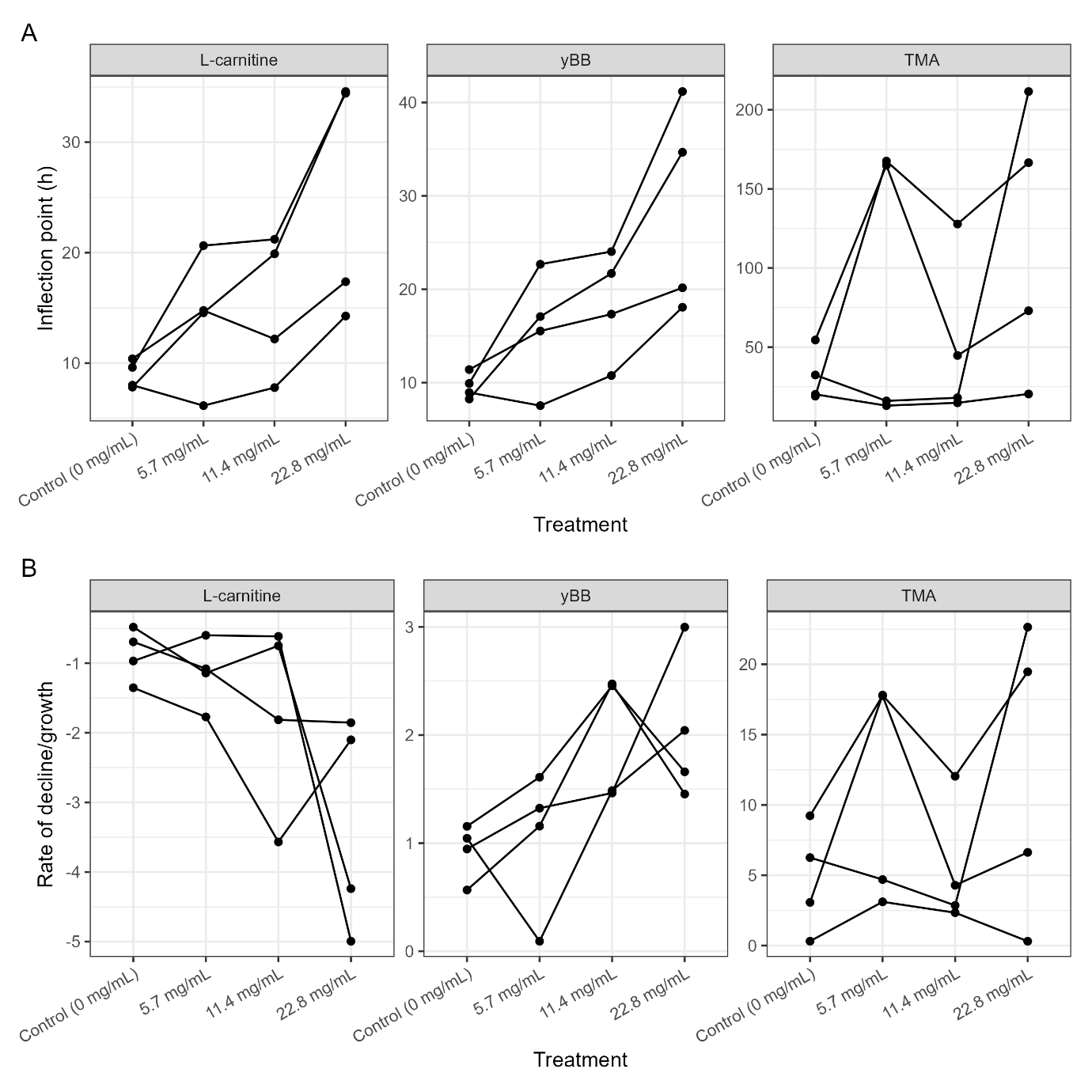


**Supplementary Figure S3**. Logistic curve model parameters corresponding to growth curves shown in figure 6. Logistic curves are characterized by the equation:

$$y=\frac{A}{1+e^{\frac{t_{50}-t}{S}}}$$

Where A corresponds to the overall asymptotic scale of the curve, and t_50_ is the midpoint.

The top panels compare the inflection point *t_50_* of the curve within donors across different pomegranate doses, which also corresponds to the point at which the curve reaches 50% of the total growth or decay. The lower panels represent how the shape of the curve *S* is affected by the pomegranate dose, with shallower growth or decay corresponding to higher absolute values.

**Supplementary Table S1**. The effect of the different pomegranate extract doses on l-carnitine metabolism delay, γ-BB growth delay, and on l-carnitine hill slope. Beta represents the time at which the concentration of the metabolites reached the mid-point (t_IC50_).

|  | l-carnitine disappearance time to midpoint, *t _IC50_* (h) | | | γ-BB accumulation time to midpoint, *t_IC50_* (h) | | |
| --- | --- | --- | --- | --- | --- | --- |
| Characteristic | **Beta** | **95% CI ^a^** | **p-value** | **Beta** | **95% CI ^a^** | **p-value** |
| (Intercept) | 9.0 | 0.59, 17 | 0.039 | 9.6 | 1.2, 18 | 0.031 |
| Treatment |  |  |  |  |  |  |
| Control | - | - | - | - | - | - |
| Pomegranate extract (5.7 mg/mL) vs Control | 5.1 | -2.7, 13 | 0.200 | 6.1 | -1.8, 14 | 0.120 |
| Pomegranate extract (11.4 mg/mL) vs Control | 6.3 | -1.5, 14 | 0.100 | 8.8 | 0.93, 17 | 0.032 |
| Pomegranate extract (22.8 mg/mL) vs Control | 16 | 8.4, 24 | 0.001 | 19 | 11, 27 | <0.001 |

^a^ CI = confidence interval.

**Supplementary Methods**

*Analysis of the pomegranate extract*

The pomegranate extract (Dermogranate®) was provided by Medinutrex (Catania, Italy) and has been derived from whole pomegranates. We estimated the quantities of the main pomegranate polyphenols in the extract using LC-MS/MS. A standard curve was prepared containing each of the polyphenol standards at different concentrations in 50% aqueous methanol, with concentrations starting at 100 µg/mL for punicalin, 50 µg/mL for punicalagin as well as for ellagic acid, and 5 µg/mL for gallic acid. A blank was included (50% aqueous methanol). Punicalagin and punicalin were first dissolved in 100% methanol before they were diluted in 50% v/v aqueous methanol. Ellagic acid was first dissolved in dimethyl sulfoxide (DMSO) and ultrasonicated for 30 minutes before it was diluted in 50% aqueous methanol. 50 mg of the Dermogranate® extract was prepared in 500 µL DMSO and then diluted 20-fold in 50% v/v aqueous methanol, reaching a final concentration of 500 µg/mL, which was prepared in triplicate. All samples were filtered through a 0.22 µm PVDF filter prior to analysis.

The Waters TQ Absolute (Wilmslow, UK), which combines UPLC and triple quadrupole mass spectrometry (MS), was used to quantify the most abundant polyphenols in the pomegranate extract (punicalagin, punicalin, ellagic acid, and gallic acid). The mobile phase consisted of 0.1% formic acid in distilled water (eluent A) and 0.1% formic acid in acetonitrile (eluent B). 1 µL of each sample was run through a Luna Omega Polar C18 100A column (100 x 2.1 mm; particle size 1.6 µm) at 35°C and a flow rate of 400 µL/min. The gradient was 3% eluent B for 5 minutes, after which eluent B was increased to 20% for 4 minutes, and then further increased to 50% for 2 minutes, lastly eluent B was increased to 95% for 1 minute before it was re-equilibrated to 3% for 2 minutes. In total, each sample ran for 15 minutes. The polyphenols were identified against the matching standard using the retention time (RT) and *m/z* [M-H]^-^. The spectra were inspected using the SYNAPT G2-Si program.

*Choice of substrate concentrations*

The use of 2 mM substrate in the experimental setup was not primarily intended to reflect physiological relevance, but rather to establish a well-defined metabolic curve. When we undertook experiments with much lower concentrations of choline/l-carnitine, the loss of substrate was so rapid that typically we only obtained a starting concentration and a 100% complete conversion data point. Therefore, to obtain multiple time points of substrate loss and appearance of intermediate/TMA, we used 2 mM. To put this into physiological context, for example, a 200 g portion of ground beef provides 176 mg of l-carnitine ^[1]^. Considering that the human colon has been estimated to hold an average volume of 561 mL (including the ascending, transverse, and descending colon) ^[2]^, this would approximate a concentration of ~1.95 mM l-carnitine assuming that all of the substrate reached the colon. In practice, this is likely to be supraphysiological for foods but entirely achievable with supplements, which are typically doses of 500-2,000 mg.

*Supplementary references*

[1] Demarquoy, J., Georges, B., Rigault, C., Royer, M.-C., Clairet, A., Soty, M., Lekounoungou, S., Le Borgne, F., Radioisotopic determination of l-carnitine content in foods commonly eaten in Western countries. *Food Chemistry* **2004**, *86*, 137.

[2] Pritchard, S. E., Marciani, L., Garsed, K. C., Hoad, C. L., Thongborisute, W., Roberts, E., Gowland, P. A., Spiller, R. C., Fasting and postprandial volumes of the undisturbed colon: normal values and changes in diarrhea‐predominant irritable bowel syndrome measured using serial <scp>MRI</scp>. *Neurogastroenterology &amp; Motility* **2014**, *26*, 124.
